# Recalcitrant folliculitis decalvans successfully controlled with adalimumab^☆^

**DOI:** 10.1016/j.abd.2024.02.001

**Published:** 2024-03-28

**Authors:** José Ramos, António Magarreiro Silva, Ana Marta António, João Alves

**Affiliations:** Dermatology and Venereology Department, Hospital Garcia de Orta EPE, Almada, Portugal

Dear Editor,

Folliculitis Decalvans (FD) is a primary cicatricial neutrophilic alopecia that affects young adults, mainly men, with a typically chronic and relapsing course. The pathogenesis remains unclear, current evidence suggests that in patients with genetic predisposition, there is an altered interrelationship between local immunity and the microbiome (mainly colonization by *Staphylococcus aureus*) culminating in chronic stimulation of T-cell homing.^1^, ^2^ Clinically, it starts with pruriginous or painful plaques in the vertex, with tufts of hairs and perifollicular crusts, which leads to the permanent destruction of hair follicular stem cell structure and subsequent replacement with fibrous tissue. Treatment of FD is challenging, characterized by prolonged courses of medications and frequent relapse after suspension.^3^ The therapeutic goals should be focused on controlling outbreaks and preventing the irreversible progression of alopecia.^2^

Herein we report a case of FD with extensive involvement in the scalp whose disease control was only obtained with adalimumab.

A 39-year-old-male with a personal history of bronchial asthma and ischemic transient stroke at 22 years of age as a complication of renovascular hypertension, was previously followed in the Dermatology department of our hospital with a diagnosis of FD, with a histopathological examination of a scalp biopsy compatible with the diagnosis (Fig. 1). Back then, in 2015, in addition to topical and intralesional corticosteroids, he was treated with various regimens of doxycycline, and rifampicin/clindamycin with initial clinical improvement, but once the medication was stopped, relapses were observed. After one year, he started missing appointments and lost follow-ups. For about five years, he had irregular appointments in private Dermatology and was treated with several cycles of doxycycline and clindamycin. In November 2021, he returned to our consultation, with clinical worsening. He was quite symptomatic, with continuous pain that affected his quality of life. He also mentioned that there had been a decreasing response to oral antibiotics in recent years. Clinical observation revealed extensive involvement of the scalp, with multiple-scarring alopecic patches with inflammatory active lesions in the borders, characterized by follicular pustules, tufting of hairs, and hemorrhagic crusts (Fig. 2A, 2B, and 2C). On trichoscopy, there was diffuse erythema, alongside capillary tufts, follicular pustules, and perifollicular scale (Fig. 2D). Laboratory tests were found to be normal. He started treatment with oral isotretinoin and azithromycin. After six months, given an absence of a response, the medication was suspended and changed to adalimumab (dosing regimen of 160 mg subcutaneously on day 1, 80 mg starting on day 15, and 40 mg weekly starting from day 29). Disease control was achieved after three months of therapy. The patient has been treated with adalimumab for the last 15 months with only one flare observed during this period, which was successfully resolved with the addition of doxycycline (already suspended). In the last appointment, he was asymptomatic with no active lesions at clinical and trichoscopy examinations (Fig. 3).

**Fig. 1:**
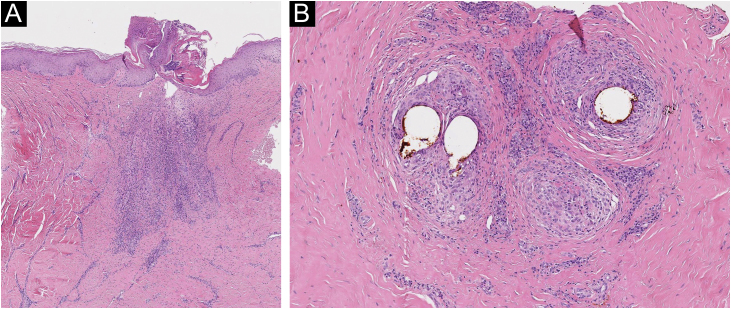
(A) In the vertical sections: a lymphohistiocytic infiltrate with neutrophils, at the perivascular and periadnexal level, with fibrosis of the dermis is observed. (B) In horizontal sections: there is a perifollicular lymphohistiocytic infiltrate with neutrophils mainly in the upper zones of the follicle with concentric perifollicular and interfollicular fibrosis, with loss of sebaceous glands. PAS and Giemsa stainings excluded microorganisms in the observed samples (Hematoxylin & eosin, ×40 [A], [B] Hematoxylin & eosin, ×100).

**Fig. 2:**
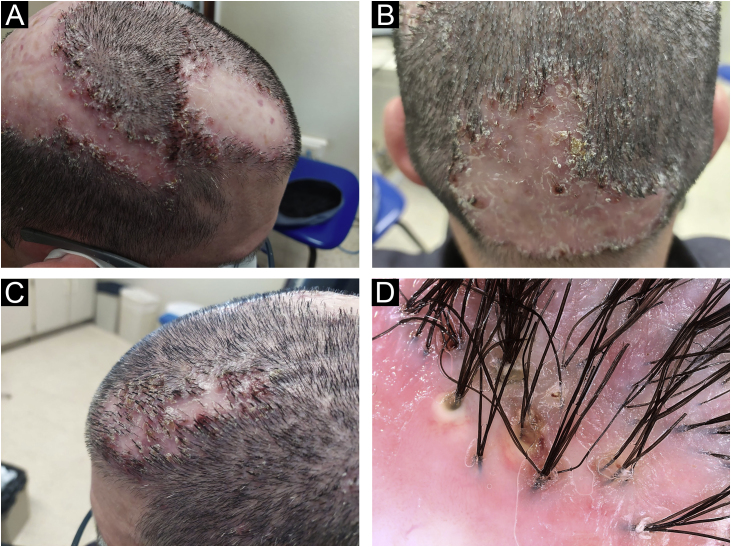
Clinical aspect (A, B, and C) and trichoscopy (D) - examination before treatment with adalimumab.

**Fig. 3:**
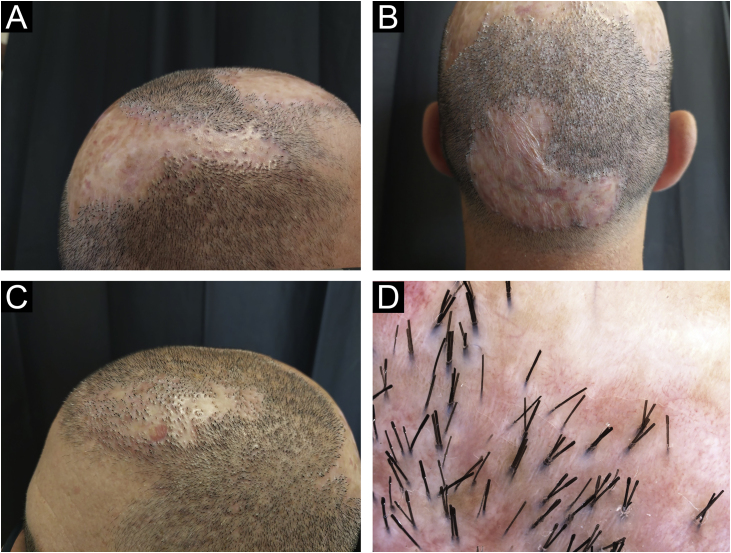
Clinical aspect (A, B, and C) and trichoscopy (D) - examination after the treatment.

Tumor necrosis factor is a cytokine whose role as a mediator of inflammatory processes has been widely described, and that is commonly encountered in neutrophilic dermatoses. The inhibition of tumor necrosis factor-α with biological treatment has been successfully used in many of these diseases, with a positive outcome.^4^, ^5^ This case illustrates the difficulties faced when treating FD and supports existing evidence, based on clinical case series, regarding the efficacy of adalimumab in the treatment of refractory FD.^6^

## Financial support

None declared.

## Authors’ contributions

José Ramos: Approval of the final version of the manuscript; preparation and writing of the manuscript; Manuscript critical review.

António Magarreiro Silva: Approval of the final version of the manuscript; manuscript critical review.

Ana Marta António: Approval of the final version of the manuscript; manuscript critical review.

João Alves: Approval of the final version of the manuscript; manuscript critical review.

## Conflicts of interest

None declared.
